# Iron-Deficiency Anemia in CKD: A Narrative Review for the Kidney Care Team

**DOI:** 10.1016/j.xkme.2023.100677

**Published:** 2023-05-25

**Authors:** Debra Hain, Donna Bednarski, Molly Cahill, Amy Dix, Bryce Foote, Mary S. Haras, Rory Pace, Orlando M. Gutiérrez

**Affiliations:** 1 Florida Atlantic University, Boca Raton, FL; 2 Detroit Medical Center Harper University Hospital, Detroit, MI; 3 Kansas City Kidney Consultants, Kansas City, MO; 4 Akebia Therapeutics Inc, Cambridge, MA; 5 Georgetown University School of Nursing, Washington, DC; 6 Satellite Healthcare, San Jose, CA; 7 University of Alabama at Birmingham, Birmingham, AL

**Keywords:** Anemia, chronic kidney disease, iron deficiency, hemoglobin, iron-deficiency anemia, iron metabolism

## Abstract

Anemia is a common complication of chronic kidney disease (CKD) and is associated with increased mortality and reduced health-related quality of life. Anemia is characterized by a decrease in hemoglobin, the iron-rich protein that the body uses for oxygen transport. Iron is required to produce hemoglobin, and disruptions in the iron homeostasis can lead to iron-deficiency anemia. Management of anemia in individuals with CKD is typically performed by a team of physicians, nurse practitioners, physician assistants, or registered nurses. Throughout the care continuum, the management can be enhanced by multidisciplinary care, and individuals with CKD can benefit from the involvement of other specialties, with dietitians/nutritionists playing an important role. However, a key area of unmet clinical need is how to assess and address iron-deficiency anemia. This review aims to provide an overview of iron-deficiency anemia in CKD and how this may be diagnosed and managed by the entire kidney care team, such as describing the mechanisms underlying iron homeostasis, the complications of iron-deficiency anemia, and the current challenges associated with its diagnosis and treatment in CKD. Opportunities for each multidisciplinary team member to add value to the care of individuals with CKD and iron-deficiency anemia are also described.

## Introduction

Anemia is a common complication of chronic kidney disease (CKD), which is associated with a significantly heightened risk of cardiovascular morbidity and higher mortality rates.^1^ Direct health care costs are higher in individuals with CKD and anemia than for those without anemia. Many individuals experience poor health-related quality of life (QoL: eg, fatigue and reduced productivity) and debilitating symptoms, such as cognitive impairment, shortness of breath, dizziness, headaches, loss of appetite, and depression.^2^, ^3^, ^4^, ^5^ The overall prevalence of anemia in CKD was estimated to be 15.4%, with the prevalence of anemia increasing as the disease advances.^1^

Anemia is clinically defined as having low levels of hemoglobin, the iron-rich protein in red blood cells (RBCs) responsible for the transport of oxygen.^6^, ^7^, ^8^ In particular, the Kidney Disease: Improving Global Outcomes (KDIGO) anemia work group guidelines define anemia in CKD as a hemoglobin concentration of <13.0 g/dL in men and <12.0 g/dL in women^9^ (hemoglobin concentrations are >13.5-17.5 g/dL in healthy men and >12.0-15.5 g/dL in healthy women).^10^

The causes of anemia in CKD are multifaceted, but anemia primarily results from erythropoietin deficiency or iron deficiency. Although an in-depth discussion of the multifactorial pathogenesis of anemia in CKD is outside the scope of this review, we refer readers to the studies by Batchelor et al,^11^ Ganz and Nemeth,^12^ and Coffey and Ganz^13^ for more detailed reviews on the topic.

Anemia in individuals with CKD is considerably undertreated, particularly in those with non–dialysis-dependent (NDD) CKD.^3^^,^^14^ In a survey of 410 individuals with NDD-CKD anemia in the United States, only 22.8% reported being treated for anemia, with the most being treated for anemia in later stages of CKD (stage 1, 12.1%; stage 2, 16.2%; stage 3, 26.5%; stage 4, 20.7%; and stage 5, 43.0%).^1^ The undertreatment of anemia in individuals with NDD-CKD also increases the need for blood transfusions.^15^^,^^16^ Among kidney transplant candidates, transfusions increase the possibility of allosensitization (ie, the development of antibodies), which may preclude candidates from being able to receive the transplant or may cause transplant rejection altogether.^9^ These examples highlight the importance of promptly diagnosing and managing anemia in this population. Because of the complexity of anemia in individuals with CKD, successful treatment requires a multidisciplinary care team. These specialists include physicians, nurse practitioners, physician assistants, registered nurses, dietitians, and pharmacists. Social workers and case managers or care coordinators may also be involved, depending on individual needs. Equipped with the right knowledge and plan of action, a multidisciplinary team can play a vital role in improving health outcomes in this population.

### Molecular Mechanisms of Iron Homeostasis

#### Overview of normal iron homeostasis

Before addressing the importance of multidisciplinary management of iron-deficiency anemia in CKD, it is important to briefly revisit the molecular mechanisms of normal iron homeostasis. Iron is a crucial physiological element with a unique ability to both receive and transfer electrons, thereby participating in several critical physiological processes, such as cellular respiration, oxygen transport, and storage. Because of the potential for excess iron to cause severe oxidative stress and tissue damage,^6^^,^^13^ iron is bound to iron transport proteins through tightly regulated iron metabolism. Iron is an essential constituent of hemoglobin molecules, which have a protein component and an oxygen-binding heme-iron complex within their α and β chains. Several hundred million hemoglobin molecules, which transport oxygen throughout the body, are contained in a single RBC.^17^

**Figure 1:**
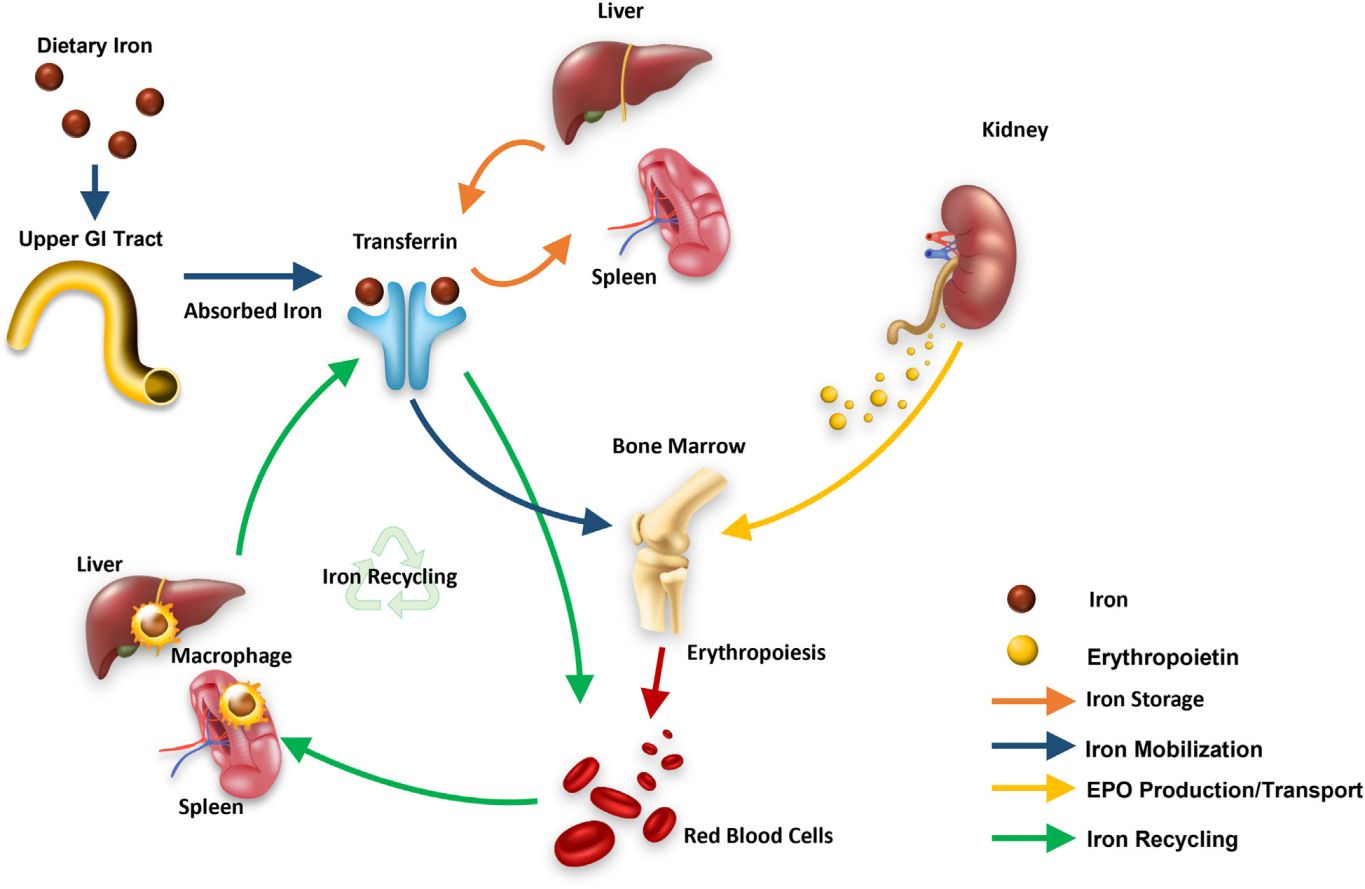
Overview of normal iron homeostasis. Iron is a key component of hemoglobin, which is used by RBCs to transport oxygen. Approximately 24 mg of iron per day is necessary for RBC production. Dietary iron is absorbed by the enterocyte; ∼1 to 2 mg must be replaced through gastrointestinal absorption per day. Iron binds to transferrin (an iron transport protein) in the blood. Transferrin delivers iron to the bone marrow (site of hemoglobin formation and RBC production). Iron not found in the circulation is largely stored in the liver and spleen bound to ferritin (an iron-storage protein). Iron is largely recycled from aged RBCs (∼120 days old), which are degraded by macrophages. Transferrin also binds to and transports the iron released from macrophages. EPO, erythropoietin; GI, gastrointestinal; RBC, red blood cells.

Dietary iron, in the form of either heme (ie, found only in meat, poultry, seafood, and fish) or nonheme iron (ie, found in plant-based foods such as grains, beans, vegetables, fruits, nuts, and seeds), is absorbed by the duodenal enterocytes (comprising the first part of the small intestine) and exported into the circulation, where it is bound to transferrin (an iron transport protein). Iron is then transported to the liver and spleen, where it is bound to ferritin (an iron-storage protein found in macrophages) or to the bone marrow, where it is used for erythropoiesis (RBC production) (Fig 1).^11^^,^^14^^,^^18^ Under normal conditions, the bone marrow generates ∼200 billion new RBCs per day to match the number of old (senescent) RBCs removed from the circulation.^19^^,^^20^ Erythropoiesis is regulated by erythropoietin (a hormone that stimulates RBC production), which is mainly produced by the kidneys and by the liver to a lesser extent.^21^^,^^22^ Iron stores are replenished when macrophages in the spleen and liver engulf and consume old RBCs, a process known as phagocytosis, ultimately recycling their iron content, which is used either for the production of new RBCs or stored for future use.^6^^,^^20^^,^^23^ The recycling of RBCs in the spleen and liver provides most of the body’s iron (20-25 mg/d),whereas dietary absorption provides only 1-2 mg/d of iron.^20^^,^^22^ Most of the body’s iron is stored in ferritin, and ∼3 mg of iron can be found circulating bound to transferrin, which must be turned over every few hours to meet the daily requirements.^21^^,^^23^

#### Hepcidin as a key regulator of iron homeostasis

**Figure 2:**
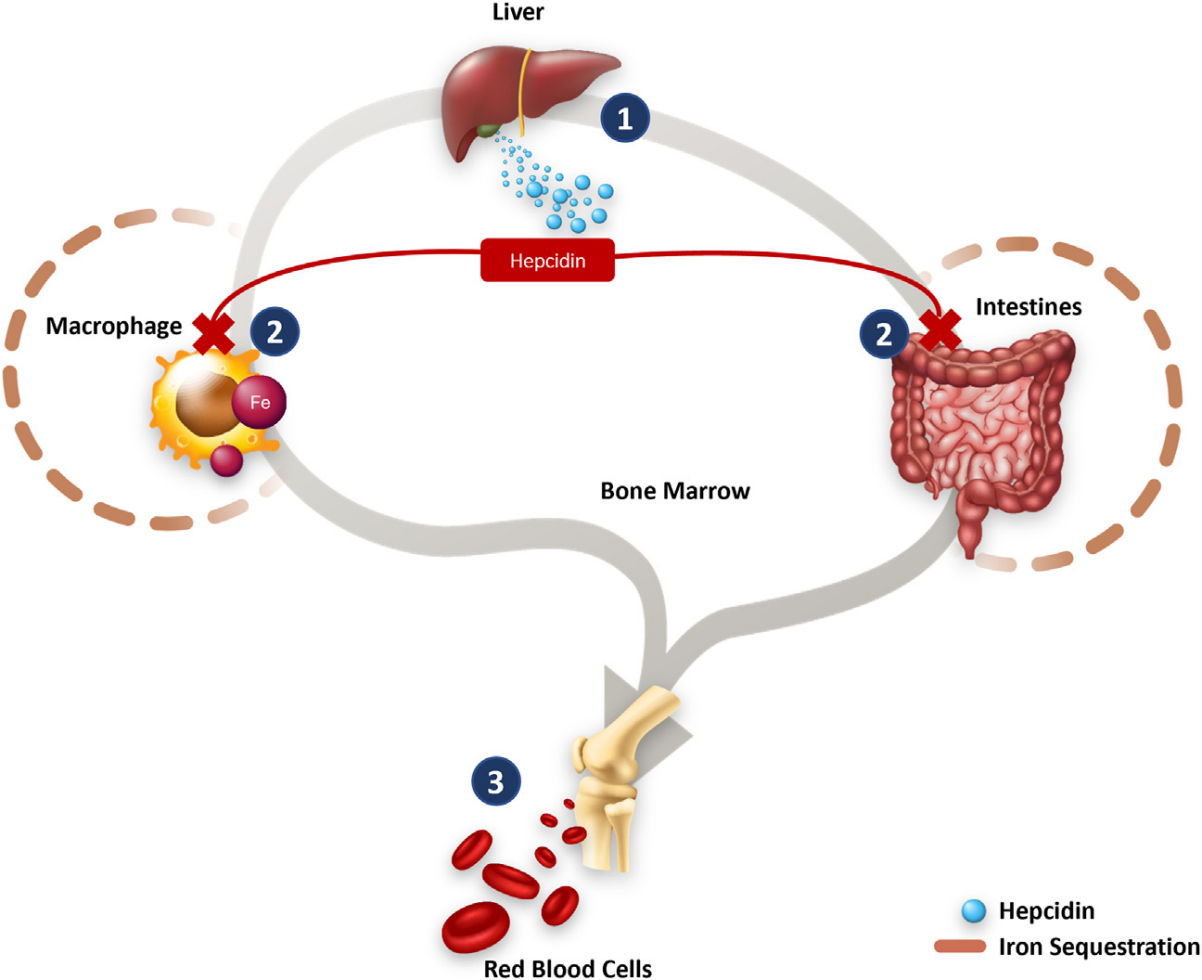
Hepcidin regulates iron absorption and iron availability. Hepcidin production is stimulated in the liver in response to increased iron uptake, inflammation, and infection (step 1); high levels of hepcidin result in limited iron availability because iron is sequestered by the intestine and macrophages (step 2) ferroportin serves as an exporter of iron into circulation. Because of high levels of hepcidin, ferroportin interacts with hepcidin, which results in endocytosis and degradation. The degradation of ferroportin leads to impaired iron absorption and release from the iron-storage sites, which results in decreased red blood cell production (step 3).

Hepcidin is the key hormonal regulator of iron homeostasis through its effects on ferroportin,^14^ a protein that exports iron from the inside of the cell to the outside of the cell. Hepcidin controls the uptake of iron from the gut and its release from the iron stores^11^^,^^13^^,^^24^ (Fig 2). Hepcidin production is stimulated by increased iron uptake, inflammation, and infection and is inhibited by iron deficiency and hypoxia (inadequate oxygen supply to cells).^11^^,^^24^^,^^25^ Hepcidin is released from the liver cells into the circulation and binds to the iron exporter ferroportin that is located on the membranes of the small intestine, macrophages, and liver cells. This binding ultimately causes the degradation of ferroportin, preventing iron export into the plasma^21^^,^^26^ and, in turn, sequestering iron in iron-storage sites (liver cells and macrophages).

### Causes of Iron Deficiency in CKD

Iron-deficiency anemia in individuals with CKD may result from functional iron deficiency, absolute iron deficiency, or both. The factors contributing to functional iron deficiency are chronic inflammation and poor hepcidin clearance seen in CKD, whereas absolute iron deficiency includes poor gastrointestinal (GI) dietary iron absorption and blood loss.^27^ Understanding the cause of iron deficiency in individuals with CKD is crucial, and this knowledge guides the most appropriate and effective plan of care.

#### Functional iron deficiency—CKD exacerbates iron deficiency

**Figure 3:**
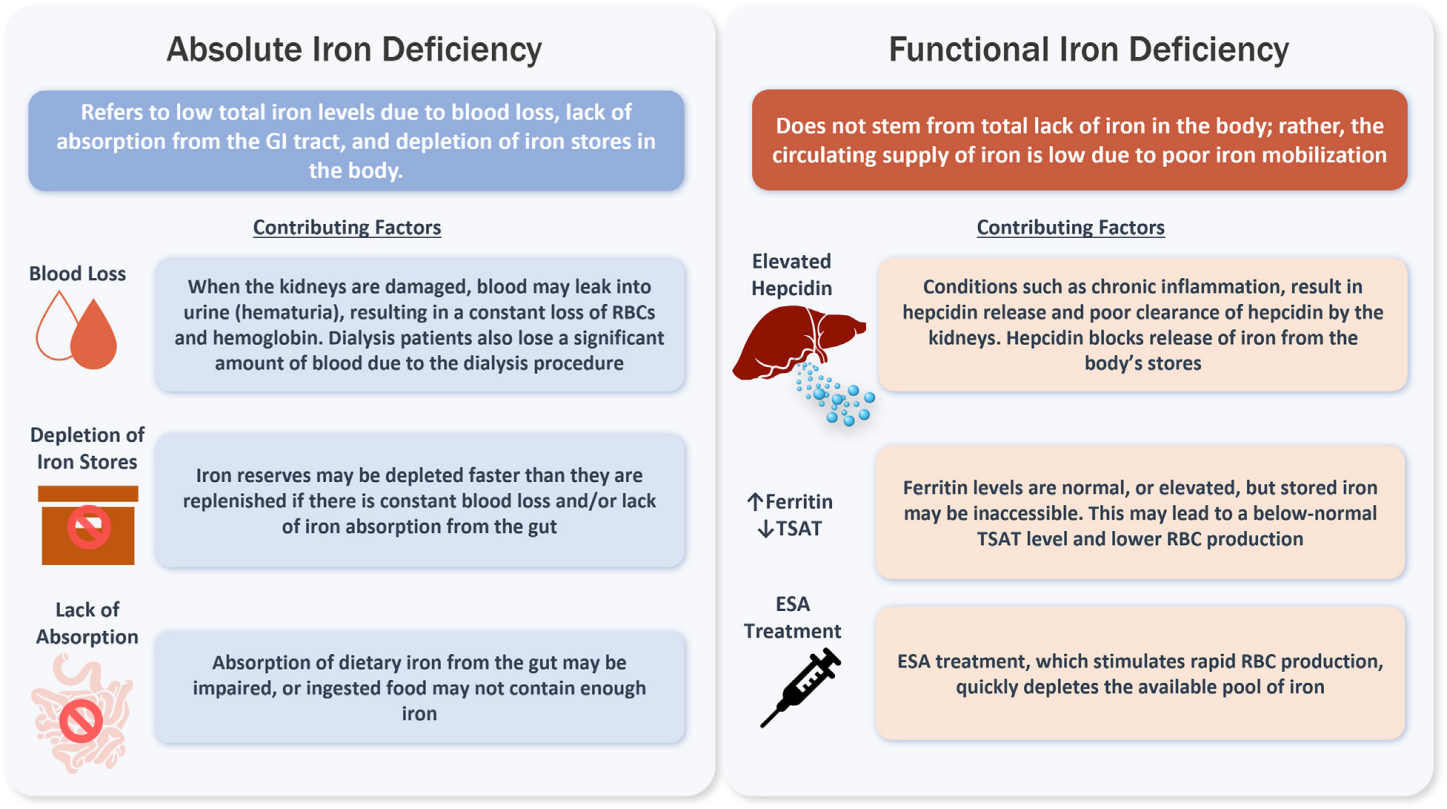
Functional versus absolute iron deficiency.^6^^,^^11^^,^^14^ Depending on its cause, iron deficiency can be categorized as absolute or functional. Both forms of iron deficiency are common in CKD. CKD, chronic kidney disease; ESA, erythropoietin-stimulating agent; GI, gastrointestinal; TSAT, transferrin saturation.

Impaired kidney function resulting from CKD can lead to reduced erythropoietin production and reduced RBC production. Erythropoiesis-stimulating agents (ESAs), such as epoetin alfa and darbepoetin alfa, which have been staple treatments for anemia in CKD, commonly cause functional iron deficiency because after each dose they transiently increase erythropoiesis to a higher rate than would occur naturally.^27^ Therefore, iron release into the circulation from iron stores is not rapid enough to provide sufficient iron to support the increased rate of erythropoiesis, causing a relative deficiency of circulating iron.^6^^,^^27^ When this occurs, it is known as functional iron deficiency.^11^ The prevalence of functional iron-deficiency anemia in individuals with CKD is high, and this can be a difficult clinical condition to manage. One way to mitigate this is through iron supplementation, which is recommended for individuals with CKD who are receiving ESAs to prevent the development of iron deficiency.^9^ In addition, CKD leads to a highly inflammatory state that can chronically elevate hepcidin levels and result in dysregulation of iron homeostasis. Hepcidin is a relatively small hormone peptide that is degraded by the kidney and excreted in the urine.^25^ Thus, increased hepcidin in CKD can result from impaired kidney clearance, which correlates with poor kidney function. Inflammatory cytokines, such as interleukin (IL)-6, also directly induce hepcidin transcription. Elevated hepcidin prevents the release of stored iron from absorptive duodenal epithelial cells, macrophages, and hepatocytes, reducing the availability of iron for erythropoiesis.^28^^,^^29^ This can cause high serum ferritin concentrations and low transferrin saturation (TSAT; defined as plasma iron divided by the total iron-binding capacity × 100), which can be characteristic of functional iron-deficiency anemia (Fig 3).^14^^,^^27^

The hepcidin-induced blockade of iron further limits responsiveness to erythropoietin because the differentiation of erythroblasts (immature RBCs) to reticulocytes (nearly mature RBCs) is an iron-dependent process.^11^ Anemia of chronic inflammation is a common cause of functional iron deficiency and can occur in inflammatory diseases other than CKD (in the absence of therapy with ESAs).^11^ Anemia from chronic inflammation may also be triggered by active infection, autoimmune diseases, cancer, hypoxia, or genetic deficiencies.^8^^,^^11^^,^^20^ In individuals with CKD and functional iron deficiency, TSAT, which reflects circulating iron, may be ≤20.0% because the bone marrow strips iron off the circulating transferrin faster than it can be replenished from the iron stores. Ferritin, which reflects stored iron, may be low or elevated (Fig 3).^9^

#### Absolute iron deficiency

The functional iron deficiency observed in CKD contrasts with absolute iron deficiency, which occurs when the total body iron stores in the bone marrow, liver, and spleen are depleted. Absolute iron deficiency in individuals with CKD is defined by low circulating and stored iron, particularly TSAT of ≤20.0% and ferritin of ≤100 ng/mL.^6^^,^^27^ Absolute iron deficiency may be a result of poor dietary iron absorption in the GI tract, malnutrition, blood loss due to GI bleeding, or frequent blood tests or hemodialysis (in kidney failure) (Fig 3).^11^^,^^27^^,^^30^^,^^31^ Individuals with dialysis-dependent CKD, particularly those treated with hemodialysis, are particularly vulnerable to iron-deficiency anemia owing to blood retained in the hemodialysis machine and tubing (as high as 2 g of iron per year).^6^^,^^32^ Medications used to treat individuals with CKD, for example, renin–angiotensin-converting enzyme inhibitors, may also disrupt erythropoiesis and inhibit dietary iron uptake, causing iron-deficiency anemia.^3^

### Iron-Deficiency Anemia and Morbidity and Mortality

Several large studies have reported that anemia in CKD is associated with a higher risk of morbidity and mortality, such as a higher prevalence or risk of cardiovascular disease, congestive heart failure, kidney failure, hospitalizations, and death.^4^^,^^6^^,^^21^^,^^33^, ^34^, ^35^ A longitudinal study of a large population (∼28,000) with CKD found that over a 5-year period, congestive heart failure, coronary artery disease, and diabetes were more prevalent in those who died and that these individuals also showed a high baseline prevalence of anemia.^33^ A separate study in men with moderate and severe CKD who were not yet treated with dialysis (N=853) found that a lower hemoglobin level was significantly associated with higher non–dialysis-dependent mortality and higher risk of kidney failure.^34^ Rates of mortality, cardiovascular complications, and kidney failure were highest in men with the most severe anemia (hemoglobin concentration of <10.5 g/dL).^34^ The same findings were reported by a separate research group that analyzed the outcomes of 5885 individuals with CKD.^4^ Cognitive impairment has also been associated with CKD, which may be due to comorbid cerebrovascular disease, which commonly occurs, and has also been linked to anemia.^36^

Anemia in CKD may also contribute to reduced QoL.^37^ In an example from a study in the United States and Canada, higher hemoglobin levels were associated with improved QoL domains of the Kidney Disease Quality of Life questionnaire in 1186 individuals with NDD-CKD stages 3-5. Because hemoglobin concentration increased from <11 to 13 g/dL, significant improvements in varied QoL domains were observed, suggesting a relationship between hemoglobin level and QoL. However, the most dramatic improvements in QoL domains occurred when hemoglobin levels increased in the groups with a baseline hemoglobin concentration of <11 g/dL and hemoglobin concentration of 11-12 g/dL. In a separate real-world, international study of 2121 individuals with NDD-CKD, hemoglobin concentration of >12 g/dL were associated with better health-related quality of life, higher odds of being physically active, reduced odds of progression of CKD, and greater survival.^38^ In addition, this study demonstrated that even moderate anemia was associated with poorer health-related quality of life, CKD progression, and mortality.^38^ Malnutrition is also a common complication of CKD that has been associated with impaired QoL and well-being^39^ in addition to its effect on iron deficiency and anemia.

**Figure 4:**
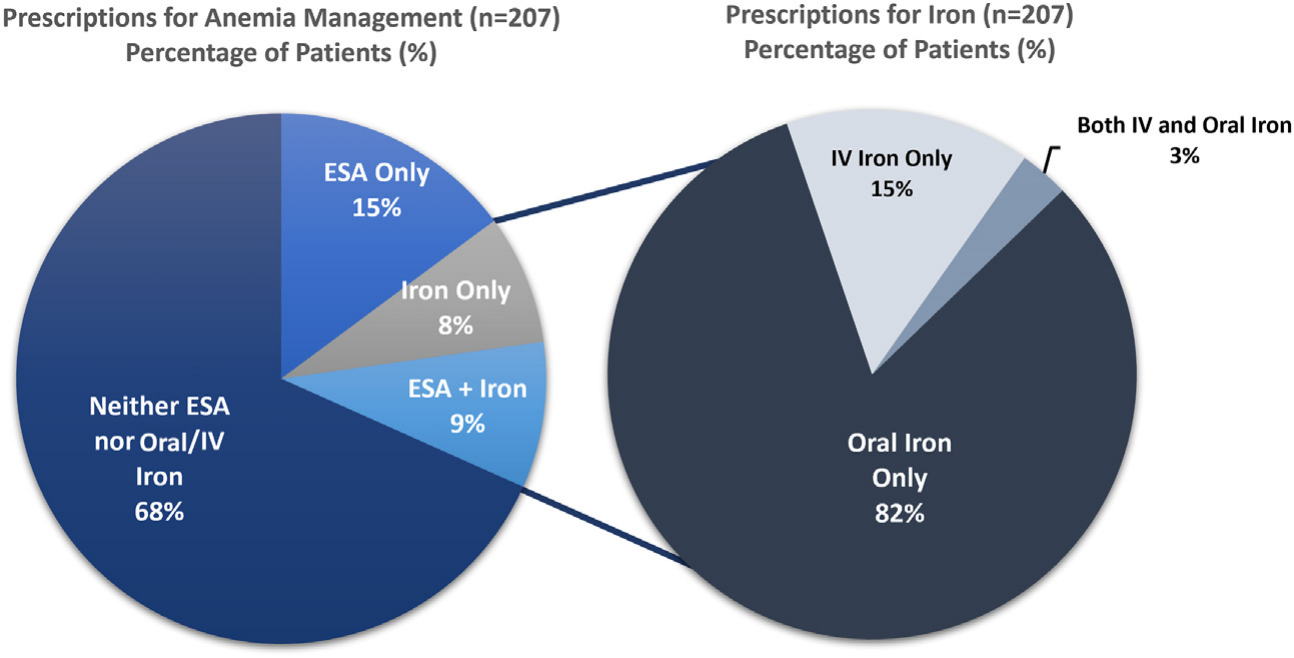
Prescriptions for anemia management in US individuals with NDD-CKD with hemoglobin concentration of <11.0 g/dL.^3^ CKD, chronic kidney disease; ESA, erythropoietin-stimulating agent; IV, intravenous; NDD, non–dialysis-dependent.

The effect of anemia in CKD on the clinical end points discussed earlier highlights the importance of optimal anemia management and the nuances that might exist in this landscape. In this context, recent data suggest that anemia in individuals with CKD may be undertreated and that there are regional differences in anemia management. For example, an international, prospective cohort study conducted by the Chronic Kidney Disease Outcomes and Practice Patterns Study (CKDopps) that was designed to describe and evaluate variation in nephrologist-led CKD practices reported that 68.0% of individuals with CKD in the United States with a hemoglobin concentration of <11.0 g/dL were not treated with iron or ESAs (Fig 4). Furthermore, the CKDopps study also found considerable differences between countries (Brazil, France, Germany, and the United States) in the management of individuals with NDD-CKD; for individuals with a hemoglobin concentration of <11.0 g/dL, <50.0% were prescribed either iron or an ESA in France and the United States.^3^ The reasons for undertreatment of iron-deficiency anemia in CKD may be due to regional, practice, and clinician variability in the monitoring of hemoglobin level and iron in those with CKD. Early findings from the CKDopps study found that a high percentage of individuals did not have their iron indices measured.^3^ A more recent analysis from the CKDopps study reported that the hemoglobin levels and iron stores are measured less frequently than the guideline recommendations across France, the United Kingdom, Brazil, and the United States, with measurement occurring more frequently in those with more advanced stages of CKD and lower hemoglobin levels.^3^ The authors of the CKDopps study noted that earlier treatment of iron-deficiency anemia in CKD would potentially prevent the rapid worsening of anemia observed in the period prelude to dialysis and may be part of a broader strategy to reduce the high mortality rates that characterize the early dialysis period.^3^

In individuals with CKD who are eligible for kidney transplant, it is vital that they are prevented from receiving transfusions or becoming transfusion-dependent because of severely low hemoglobin levels. Transfusions for the treatment of anemia in CKD can increase the risk of allosensitization. Undertreatment of iron-deficiency anemia in CKD may also result from challenges associated with the reliable measurement of iron, poor absorption of iron across the GI tract, tolerability issues related to iron supplementation, and accurate measurement of response to iron supplementation, resulting in persistent challenges in the overall management of these individuals.

#### Assessment of anemia and iron status

**Figure 5:**
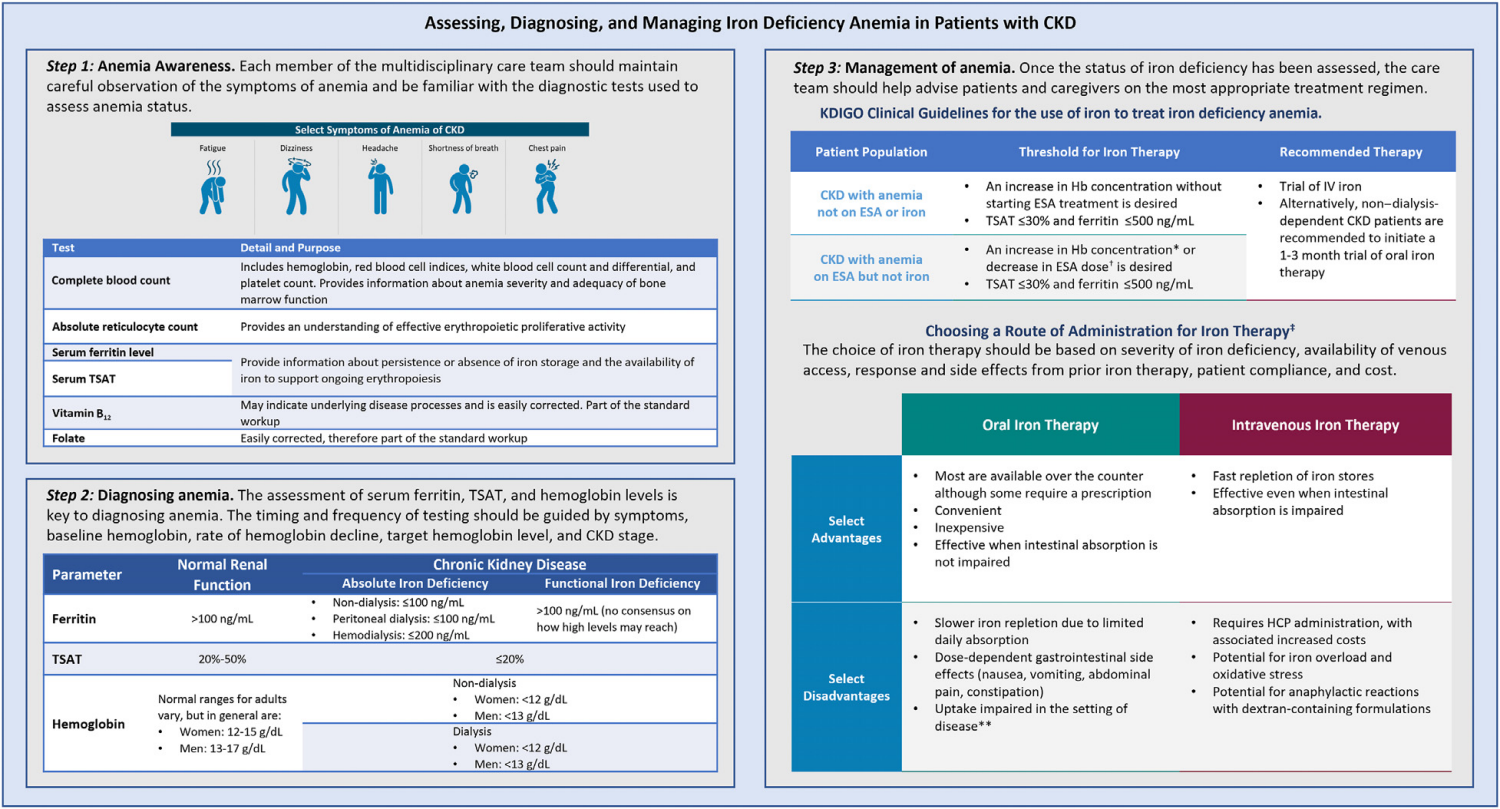
A flow chart for the assessment, diagnosis, and management of iron-deficiency anemia in patients with CKD^8^^,^^9^^,^^40^ ∗For individuals with NDD-CKD who have hemoglobin concentrations of <10.0 g/dL, KDIGO recommends that the decision to initiate ESA therapy be individualized based on the rate of decrease of the hemoglobin concentration, previous response to iron therapy, the risk of needing a transfusion, the risks related to ESA therapy, and the presence of symptoms attributable to anemia. For individuals with CKD, KDIGO suggests using ESA therapy to avoid hemoglobin concentration decreasing to <9.0 g/dL by initiating ESA therapy when hemoglobin concentration is 9.0-10.0 g/dL. ^†^On the basis of individual symptoms and overall clinical goals such as avoidance of transfusion and improvement in anemia-related symptoms and after exclusion of active infection and other causes of ESA hyporesponsiveness. ^‡^Benefits of iron therapy should be balanced with risks of harm in individuals (eg, anaphylactoid/other acute reactions and unknown long-term risks). ∗∗Disease settings of celiac disease, anemia of chronic disease, and autoimmune gastritis. CKD, chronic kidney disease; ESA, erythropoiesis-stimulating agent; IV, intravenous; KDIGO, Kidney Disease Improving Global Outcomes; NDD, non–dialysis-dependent; TSAT, transferrin saturation.

The diagnosis of iron-deficiency anemia is essential to ensure prompt treatment to correct the deficiency and improve the accompanying anemia. All individuals with CKD, particularly those with an estimated glomerular filtration rate (eGFR) of <60 mL/min/1.73 m^2^ (stage ≥3 CKD), should be screened for anemia as part of their initial evaluation after CKD diagnosis. The screening includes measurements of proteins involved in iron metabolism, with serum ferritin, hemoglobin level, and TSAT being the main tests used.^6^^,^^9^ Tests commonly used for the evaluation of anemia and their purposes are summarized in Fig 5.^40^

The members of the patient care team can help identify the various factors that may contribute to anemia in CKD by using a person-centered approach.^41^ Although the hematologic and iron status should be evaluated, the screening should also encompass baseline assessments such as current medications (including all supplements), blood pressure, pulse, and common symptoms of anemia (eg, fatigue, dizziness, headaches, shortness of breath, and chest pain).^41^ Dietitians can assess nutritional status, such as appetite, GI symptoms, weight change, and 24-hour dietary recall. If there is no dietitian on staff at the clinical practice, referral of individuals with CKD to a dietitian should be considered an important part of care.^42^

TSAT, serum ferritin, and hemoglobin levels are most commonly used to evaluate iron-deficiency anemia in clinical practice. The laboratory parameters defining iron-deficiency anemia differ between individuals with CKD with absolute or functional iron-deficiency anemia and those with normal kidney function (Fig 5). CKD management guidelines recommend that the timing and frequency of iron deficiency assessment should be guided by symptoms, baseline hemoglobin level, rates of hemoglobin decline, target hemoglobin level, and CKD stage.^9^ These recommendations are summarized in Fig 5.

An individual with CKD presenting with the clinical signs and symptoms of iron-deficiency anemia should undergo testing for iron deficiency. Therefore, all members of the multidisciplinary care team must be able to recognize these changes throughout the care continuum so that treatment can be pursued.^31^ However, the nonspecific nature of symptoms of anemia, such as fatigue and headache, means that members of the care team must be attuned to any changes in clinical indicators over the course of an individual’s care, and communication regarding findings between team members is key. Dietitians have very specialized knowledge and can assess for changes in symptoms, diet or appetite, energy levels, use of supplements, and nutritional and functional status; any findings may prompt an assessment of iron deficiency.^43^ Assessment may be performed by using validated, evidence-based tools, and dietitians should reassess nutritional status after the initial consultation as part of an ongoing, dynamic aspect of care. Social workers may take note of changes in appetite or food access that can affect nutrition and potentially trigger or worsen iron deficiency. Social workers can also leverage their training and tools to assess for depression, which may be associated with anemia. Pharmacists may pay particular attention to any changes in prescribed medications. For anyone involved in the care of individuals with CKD, a physical examination of the individual, such as looking for specific changes in nails, hair, conjunctiva, and oral mucosa, may aid in the diagnosis of deficiency of iron, folate, or vitamin B_12_. Pica behavior (craving and consuming substances that have no nutritional value, such as ice, clay, soil, chalk, or paper) has also been associated with iron-deficiency anemia, and evaluation for pica behaviors should be routinely included as part of the overall assessment.

### Treatment Options for Iron-Deficiency Anemia in CKD

It is important to diagnose iron deficiency in individuals with CKD because treatment can readily correct the associated anemia. Iron supplementation (either oral or intravenous) and/or ESA therapy are generally accepted as the standard of care for iron-deficiency anemia in CKD (correctable causes of anemia, such as iron deficiency, should be addressed before ESA initiation).^6^^,^^9^^,^^44^, ^45^, ^46^ Iron supplementation is recommended in CKD to treat iron deficiency, prevent its development in ESA-treated individuals, and support ESA utilization and dosing for those requiring ESA therapy. There are several types of oral iron supplements available for the treatment of iron deficiency; iron-only supplements are typically available in the form of ferrous or ferric salts. Heme-iron preparations are a newer type of oral iron supplement derived from bovine hemoglobin that are also available,^47^ but dietitians should be aware that these may not be suitable for vegans who may only wish to take supplements from nonanimal sources.

Guideline recommendations from KDIGO are summarized in Fig 5. These include recommendations for iron supplementation both with and without ESA therapy. Of importance, evidence from clinical trials does not support normalizing hemoglobin level with ESA treatment based on reported worse outcomes compared with lower targets, such as mortality and cardiovascular events. Therefore, most guidelines recommend a hemoglobin target range of 10-12 g/dL when treating with ESAs and avoidance of hemoglobin concentrations >13 g/dL for all adults.^3^^,^^9^^,^^41^ For iron supplementation with ESA therapy, the KDIGO guidelines recommend that supplemental iron should be administered to maintain serum ferritin levels >200 ng/mL in individuals with CKD stage 5 treated with hemodialysis and >100 ng/mL in those with NDD-CKD stage 5 treated with peritoneal dialysis with TSAT of >20% in all individuals with CKD.^9^ KDIGO guidelines do not recommend routine use of iron supplementation in patients with TSAT  >30% or serum ferritin  >500 ng/mL (>500 mg/L).^9^ Since this 2012 guidelines, there has been recognition that many individuals who are in an inflamed state and exhibit a hepcidin-induced blockade to iron may have altered iron metabolism that results in seemingly high serum ferritin (eg, >500 ng/mL) but low TSAT (eg, <15%), and thus, some guidelines suggest a maximum serum ferritin level of 800 ng/mL.^48^^,^^49^ Currently, there is a lack of consensus regarding the best parameters to estimate body iron stores to guide treatment goals.^40^ Experts agree that identifying these parameters is a high priority for future research.^40^

When initiating treatment for iron deficiency, the multidisciplinary care team can help advise individuals and caregivers on the most appropriate iron formulation, dosing, and administration schedule based on the patient’s needs and dietary preferences and address other comorbidities that may be present. Pharmacists can help identify issues related to polypharmacy and how that may affect iron supplementation.^50^ Dietitians and nutritionists become particularly important because most individuals with CKD will require dietary modifications to maintain optimal nutritional status, which usually requires re-evaluation over the course of CKD.^42^^,^^51^ These members of the care team can educate and support individuals with strategies to minimize the GI side effects that can occur with oral iron administration, while maximizing iron absorption.^52^ Guidance on adequate dietary fiber intake can also improve gut motility, manage constipation, and promote overall gut health, which is an important consideration for people with CKD in general.^51^ When folate and vitamin B_12_ deficiency contribute to the development of anemia in CKD, these vitamins can be replenished by diet and/or supplementation.^42^ Dietitians involved in the care of individuals with iron-deficiency anemia in CKD can offer nutrition and diet counseling about good sources of food rich in these nutrients^53^^,^^54^ and recommend supplementation as appropriate.

### Education in the Management of Iron-Deficiency Anemia in CKD

Each member of the multidisciplinary care team plays a vital role in educating individuals about anemia in CKD and its management. It is important that family members, care partners, or other supporters also receive education on aspects of healthy kidney function, kidney disease, common comorbidities, and other potential complications of CKD beyond anemia. Care partners and advocates should be advised to monitor for these signs, symptoms, and risk factors. Supporters should understand the basics of nutrition and pharmacological regimens (including the importance of taking medications as prescribed and potential adverse events, such as GI upset with oral iron supplementation) and the importance of physical activity.^49^^,^^55^^,^^56^

Educational resources may include focused lectures, pamphlets, and videos. Individuals with CKD can be encouraged to become actively involved in their own care and in shared decision-making by using regular telephone or in-person touch points, adoption of technology-based apps, and other self-management tools.^51^ The potential benefits of patient education related to kidney function, complications, treatment options, interventions, and lifestyle are supported by findings from a trial of 297 participants with CKD; the 149 participants who received psychoeducational intervention showed a significantly longer time to dialysis therapy compared with the 148 participants receiving usual care.^57^

In addition, involving patients and families in discussions regarding goals of care can lead to positive health outcomes, such as improved overall well-being and QoL. A systematic review of 26 studies involving more than 5000 individuals with CKD found that the most effective teaching sessions for improving individual outcomes were those that included families and multidisciplinary teams.^58^

## Conclusions

There is an ongoing opportunity to improve the management of iron-deficiency anemia in individuals with CKD, a condition that is undertreated and associated with increased mortality and reduced QoL. It is important to diagnose iron deficiency in individuals with CKD because treatment can readily correct the associated anemia. Iron supplementation is widely used to manage iron-deficiency anemia in CKD, and members of the multidisciplinary care team should be aware of the specific considerations when using this treatment. The multidisciplinary care team plays a critical role in the care of individuals with iron-deficiency anemia in CKD and has the potential to significantly improve outcomes for those who are in their care.
